# The percentage of Epidermal Growth Factor Receptor (EGFR)-mutated neoplastic cells correlates to response to tyrosine kinase inhibitors in lung adenocarcinoma

**DOI:** 10.1371/journal.pone.0177822

**Published:** 2017-05-16

**Authors:** Dario de Biase, Giovenzio Genestreti, Michela Visani, Giorgia Acquaviva, Monica Di Battista, Giovanna Cavallo, Alexandro Paccapelo, Alessandra Cancellieri, Rocco Trisolini, Roberta Degli Esposti, Stefania Bartolini, Annalisa Pession, Giovanni Tallini, Alba A. Brandes

**Affiliations:** 1 Department of Pharmacy and Biotechnology (Dipartimento di Farmacia e Biotecnologie) - Molecular Diagnostic Unit, Azienda USL di Bologna, University of Bologna, Bologna, Italy; 2 Department of Oncology, AUSL Bologna – IRCCS Institute of Neurological Sciences, Bologna, Italy; 3 Department of Medicine (Dipartimento di Medicina Specialistica, Diagnostica e Sperimentale) - Molecular Diagnostic Unit, Azienda USL di Bologna, University of Bologna School of Medicine, Bologna, Italy; 4 Anatomic Pathology Unit, AUSL Bologna, Maggiore Hospital, Bologna, Italy; 5 Interventional Pulmology, Sant'Orsola, Malpighi Hospital, Bologna, Italy; Universita degli Studi di Napoli Federico II, ITALY

## Abstract

**Background:**

Epidermal Growth Factor Receptor (*EGFR)* molecular analysis is performed to assess the responsiveness to Tyrosine Kinase Inhibitors (TKIs) in patients with Non-Small Cell Lung Cancer (NSCLC). The existence of molecular intra-tumoral heterogeneity has been observed in lung cancers. The aim of the present study is to investigate if the percentage of mutated neoplastic cells within the tumor sample might influence the responsiveness to TKIs treatment.

**Material and methods:**

A total of 931 cases of NSCLC were analyzed for *EGFR* mutational status (exon 18, 19, 20, 21) using Next Generation Sequencer. The percentage of mutated neoplastic cells was calculated after normalizing the percentage of mutated alleles obtained after next generation sequencer analysis with the percentage of neoplastic cells in each tumor.

**Results:**

Next generation sequencing revealed an *EGFR* mutation in 167 samples (17.9%), mainly deletions in exon 19. In 18 patients treated with TKIs and with available follow-up, there was a significant correlation between the percentage of mutated neoplastic cells and the clinical response (P = 0.017). Patients with a percentage of mutated neoplastic cells greater than 56%, have a statistical trend (P = 0.081) for higher Overall Survival (26.3 months) when compared to those with a rate of mutated neoplastic cells lower than 56% (8.2 months).

**Conclusions:**

The percentage of *EGFR*-mutated neoplastic cells in the tumor is associated with response to TKIs. A “quantitative result” of *EGFR* mutational status might provide useful information in order to recognize those patients which might have the greatest benefit from TKIs.

## Introduction

The adenocarcinoma tumor subtype accounts for about the 40% of all Non-Small Cell Lung Cancer (NSCLC) [1]. Molecular tests, such as analysis of Epidermal Growth Factor Receptor (*EGFR*) mutations (exons 18, 19, 20, 21) and Anaplastic Lymphoma Kinase (*ALK*) fusion gene, are prescribed in non-squamous NSCLC to determine the responsiveness to Tyrosine Kinase Inhibitors (TKIs) or ALK inhibitors, respectively [2]. *EGFR* mutations are approximately present in 10% of lung adenocarcinoma in Caucasian population [3–12] and TKIs based therapy is strongly recommended as first-line treatment in presence of these gene markers [13–22].

For this reason, *EGFR* mutations are crucial biomarkers to select patients for TKIs based treatment, and guidelines for molecular diagnosis have been outlined by oncologic societies both in Europe and in the United States [2, 23]. The greater part of all activating mutations that confer sensitivity to TKI (up to 80–90%) are either deletions in *EGFR* exon 19 or the p.L858R mutation (exon 21), but a variety of activating *EGFR* mutations can also occur (e.g. p.G719X in exon 18) [23].

Patients with non-squamous NSCLC harboring *EGFR* activating mutations or clinical features that suggest their presence, have been enrolled in randomized clinical trials where TKIs were compared to platinum-based chemotherapy in first-line treatment settings: results have clearly shown that TKIs improve prognosis and quality of life of patients when compared to traditional chemotherapy [13–18].

In spite of the high clinical evidence to employ TKIs (afatinib, erlotinib and gefitinib) in the early phases of the treatment of patients with advanced NSCLC harboring sensitive mutations, the duration of the clinical response is variable, and about 20% of patients undergoes tumor progression during TKI therapy. Well known explanations for this “resistance” are: i) molecular alterations in genes other than *EGFR* (e.g additional mutations downstream of *EGFR* along the MAPK/Kinase pathway) [24]; ii) mutations of *EGFR* conferring resistance (e.g *EGFR* p.T790M) [25]. An additional explanation may be the *EGFR* mutation heterogeneity within the tumor [22]. In this last instance, assessment of *EGFR* mutation heterogeneity in NSCLC may recognize those patients with *EGFR* mutations that might benefit most from TKI therapy. Next Generation Sequencing (NGS), that allows quantitative assessment of mutated alleles performed in lung [26, 27], gastrointestinal tract [28], pancreatic [29, 30], thyroid [31, 32], and renal tumors [33], has demonstrated the existence of heterogeneity of the driving molecular alterations, not only within the primary tumor, but also between the primary and its metastasis [29, 31]. Recently, Bria *et al*. showed a relevant relationship between the heterogeneity of *EGFR* mutations in NSCLC and duration of clinical response after TKI treatment: patients with a high proportion of *EGFR* mutated alleles responded better to TKIs [26].

This study investigates for the first time whether, not only the frequency of *EGFR* mutated allele, but also the percentage of *EGFR* mutated neoplastic cells has an influence on the response to TKIs.

## Material and methods

**Fig 1:**
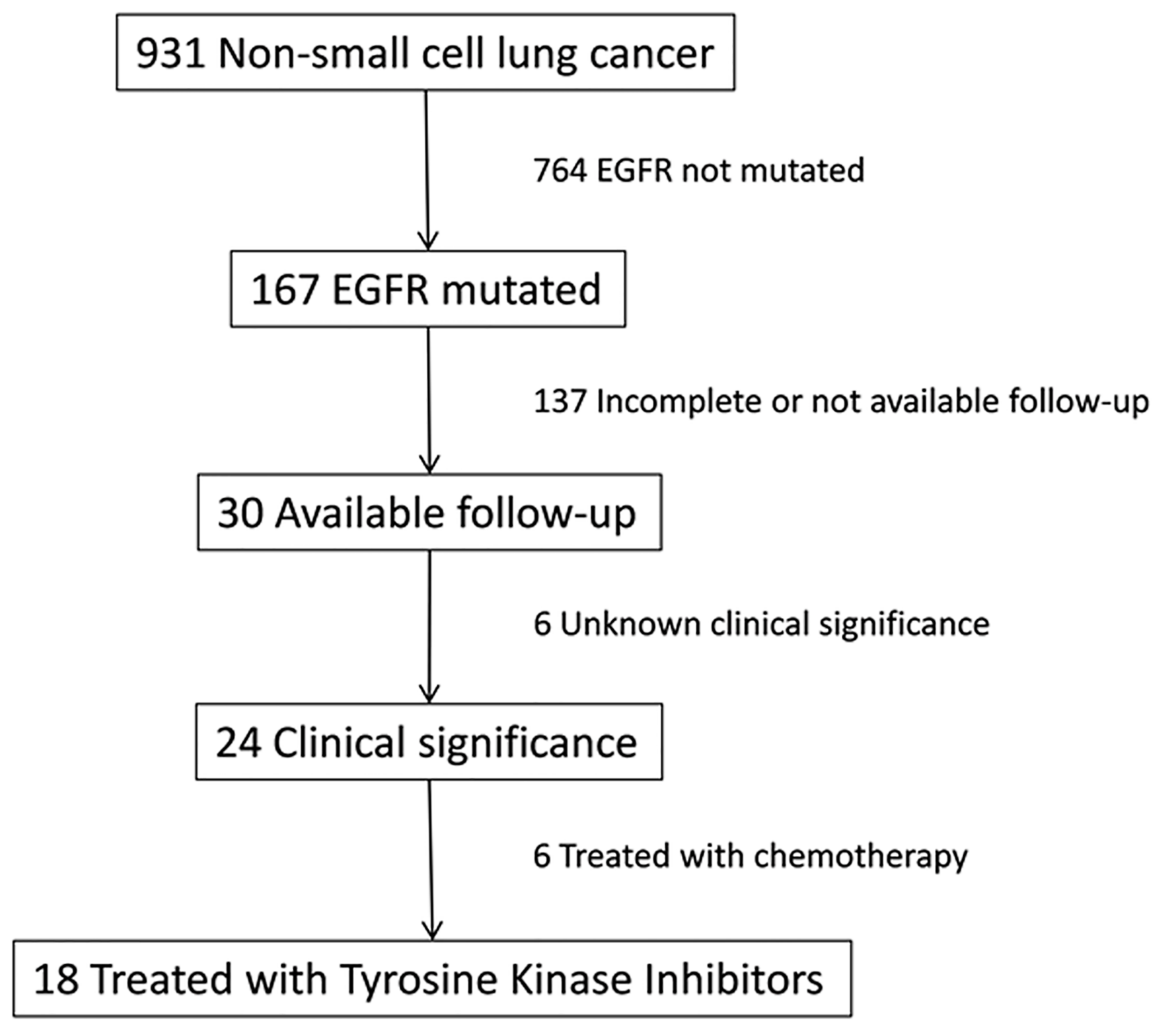
Flow chart of the cases analyzed.

Overall, a total of 931 cases of NSCLC were analyzed for *EGFR* mutational status (exons 18, 19, 20, 21) (Fig 1).

To address if *EGFR* mutation heterogeneity could influence the response to TKIs, the percentage of *EGFR*-mutated alleles was evaluated using a high sensitive approach (NGS: 454 GS-Junior, Roche), previously used to quantify mutated allele percentages of tumor samples.

All the samples were collected as part of routine diagnostic protocols and retrieved from the archives of Anatomic Pathology of Bellaria-Maggiore Hospitals (Azienda USL Bologna, Italy) and collected during routine clinical care.

Biological assessment was performed in the Molecular Diagnostic Unit, Azienda USL di Bologna. Diagnoses were centrally reviewed by two pathologists (AC, GT) and classified according to WHO criteria [1]. In 18 cases with advanced NSCLC with mutated *EGFR* treated in first-line with TKIs (15 with erlotinib and 3 with gefitinib) follow-up (FU) data was available because patients have been referred to Medical Oncology Bellaria Hospital (AUSL Bologna, Italy) (Fig 1).

The study was approved by Ethic Committee of Azienda Sanitaria Locale di Bologna (number of study CE 16013, protocol number 234/CE of 22nd March 2016, Bologna, Italy). The ethics committee waived the requirement for patient written consent. EGFR mutational analysis is part of the routine diagnostic workup of patients with NSCLC lesions treated at Azienda USL di Bologna and by necessity the authors had access to information identifying the patients. All information regarding the human material used in the study was managed using anonymous numerical codes.

### *EGFR* mutational analysis

DNA was extracted starting from one or two cytological smears (depending on the total amount of neoplastic cells in the analyzed specimens) and from one or two 10μm-thick FFPE slides. Area of interest was scraped using a sterile blade according to the pathologist selection.

DNA from cytological smears was extracted using Epicentre MasterPure DNA purification kit (Epicentre, Madison, WI, USA) according to manufacturer’s indications. DNA from FFPE samples was extracted using the QuickExtract DNA Extraction Solution (Epicentre, Madison, WI, USA).

*EGFR* analysis was performed using previously described primers and protocol.[27] In 129 (77.2%) specimens also *KRAS* (exons 2 and 3) mutational analysis was performed, as previously described [34, 35]. Only nucleotide variations observed in both strands were considered for mutational call. Ambiguous base calls associated to stretches of homopolymers four-base-pair or longer were not considered mutated due to the limitations of the pyrosequencing chemistry that is used by 454-NGS [36]. Based on previously published data [27, 37], we established as criteria to define a sample mutated the identification of the mutation in at least 10 reads *and* in at least 1% of the total number of reads analyzed.

### Percentage of mutated neoplastic cells

The proportion of neoplastic cells was expressed as a percentage after evaluation by two pathologists (AC, GT). As the estimate of this percentage is critical for the study it was carefully analyzed as follows. Two types of specimens were analyzed: formalin fixed paraffin embedded (FFPE) tissue specimens and Papanicolau (PAP) stained cytology slide smears obtained after fine needle aspiration of the tumor mass. For FFPE specimens, the tumor area marked on the hematoxylin and eosin (HE) section used as DNA extraction control was evaluated microscopically. As this control section was the last cut after four 10um-thick sections, the proportion of neoplastic cells was also evaluated on the original HE section used for the histopathological diagnosis. The tumor cell percentage difference between these two HE sections was always <10%, and the area marked on the DNA extraction control HE to guide the selection of the material scraped for DNA analysis was used to evaluate the percentage of neoplastic cells.

For PAP stained cytology slides, the percentage of neoplastic cells was evaluated on the entire surface area marked to scrape cellular material for DNA analysis.

To avoid the bias due to the larger size of tumor cells, the microscopic analysis of tumor cell content was always based on the evaluation of tumor cell nuclei vs. the number of nuclei of non-neoplastic cells (the surface areas occupied by the tumor cells vs. non-tumor cells was not taken into account). The two pathologists evaluated the percentage of neoplastic cells independently. The greatest difference in the neoplastic cell content among the two pathologists was ±10% (average absolute difference: 8%), and the mean percentage values for each case was used for the study.

The percentage of mutated alleles obtained using NGS was normalized to the proportion of neoplastic cells in each tumor using the following formula [32]:

Percentage of mutated neoplastic cells=[(MR * 2)/X] * 100

where MR is the percentage of mutated reads according to NGS and X is the estimated percentage of neoplastic cells. For all cases the tumor was postulated to be euploid.

### Evaluation of clinical response to TKI therapy

Restaging exams have been planned as daily clinical practice and the response rate was evaluated using RECIST criteria [38]. We divided patients based on the responsiveness to TKIs in 4 categories: complete response (CR), partial response (PR), stable disease (SD) and progression of disease (PD). Progression-Free Survival (PFS) was calculated from the time of diagnosis to the date of the progression of disease documented by clinical exam or radiological assessment. Overall Survival (OS) was defined as the time from the initial diagnosis until death.

### Statistical analysis

Data are reported as means, medians, ranges and frequencies. Survival data (median survival times with 95% confidence interval) were computed by the means of the Kaplan-Meier procedure. Spearman’s correlation, Kruskal-Wallis test, Breslow-Wilcoxon test and Cox proportional hazards model were applied. Receiver–operating characteristics (ROC) curves were plotted. The area under the ROC curve (AUC) was computed together with the 95% CI. The best cut-off was calculated using the method of maximization of the Youden’s index [39].

The SPSS (Version 13.0 for Windows; SPSS Inc., Chicago, IL, USA) was used as a statistical package. Two-tailed P values less than 0.05 were considered significant.

## Results

### *EGFR* mutational analysis and clinical responses

NGS revealed *EGFR* mutations in 167 (17.9%) patients of whom 94 (56.3%) females and 73 (43.7%) males, aged from 33 to 87 years (mean: 68.3 years). Specimens have been obtained from the lung primary in 111 (66.5%) and in 56 cases (33.5%) from tumor metastases (brain, bones, lymph-nodes). The 167 mutated samples were 82 (49.1%) FFPE tissue specimens and 85 (50.9%) cytology specimens.

The mean of analyzed reads in the 167 *EGFR* mutated samples was 936 (coverage: 115x -3,080x), the mean of mutated alleles in the 167 samples was 25.2% (range: 2% - 90%). No differences in the clonal distribution of EGFR mutations were observed. Specifically, we did not identify any statistical difference in the percentage of mutated neoplastic cells with G719X vs. Exon 19 Deletion vs. Exon 20 insertion vs. p.L858R vs. p.L861X (p = 0.6636). No correlation was observed between patients age and percentage of mutated neoplastic cells (r^2^ = 0.040, p = 0.607). *EGFR* mutations are summarized in Table 1. More than one mutational event was identified in 26 cases (15.6%): 6 cases had double *EGFR* mutations, in 20 cases a *KRAS* mutations co-existed with *EGFR* mutation.

**Table 1:** Type of detected *EGFR* mutations in the 167 mutated specimens.

*EGFR* Mutation	Exon	Number of mutations (%)	% range of mutated allele	Notes
**G719X** ^a^	18	13 (7.8)	4–90	
p.G719A (c.2156G>C)		9 (69.2)	9–90	
p.G719S (c.2155G>A)		4 (30.8)	4–33	
**Del Ex19**	19	85 (50.9)	2–90	
p.E746_A750delELREA ^b^ (c.2235_2249del15)		42 (49.4)	2–90	
p.E746_S752delELREATS (c.2236_2256del21)		4 (4.7)	2–39	
p.E746_T751delELREAT ^c^ (c.2235_2252del18)		8 (9.4)	2–75	
p.K745_A750delKELREA (c.2234_2248del15)		4 (4.7)	5–55	
p.L747-A750delLREA (c.2239_2248del10)		4 (4.7)	4–52	p.L747_A750delLREAinsP (n = 2); p.L747_A750delLREAinsS (n = 1)
p.L747_E749LRE (c.2239_2247del9)		5 (5.9)	5–80	p.L747_E749 + p.K745E (n = 2)
p.L747_P753delLREATSP (c.2238_2258del21)		7 (8.2)	2–55	p.T747_P753 + p.A755D (n = 1)
p.L747_S752delLREATS (c.2238_2256del19)		6 (7.1)	2–45	
Other Ex19 deletions		5 (5.9)	3–88	p.R748_A750delREA+p.L747F (n = 1); p.L747_T751delLREAT (n = 2); p.R745_A750delRELREA (n = 2)
**Ex20 insertion**	20	5 (3.0)	21–62	
**p.T790M** ^c^^,^^d^ (c.2369C>T)	20	2 (1.2)	53–60	
**p.L858R** ^b^^,^^d^^,^^e^ (c.2573T>G)	21	32 (19.2)	2–65	
**p.L861X**	21	8 (4.8)	3–60	
p.L861Q (c.2582T>A)		6 (75.0)	3–60	
p.L861P (c.2582T>C)		2 (25.0)	5–9	
**Other Mutations** ^e^	18–21	28 (16.8)	2–55	

^a^ In two sample a double mutation p.G719X + p.E709A was observed;

^b^ In one sample a double mutation DEL E746_A750 + p.L858R was observed;

^c^ In one sample a double mutation DEL E746_T751 + p.T790M was observed;

^d^ In one sample a double mutation p.L858R + p.T790M was observed;

^e^ In one sample a double mutation p.L858R + p.E709A was observed.

In 3 of the 6 cases with double *EGFR* mutations the discrepancy in the proportion of reads carrying the two mutations was divergent (>10%), implying the presence of two different *EGFR*-mutated clones. In the remaining cases, it is likely that mutations occurred in the same neoplastic cell clone: in two cases the percentage of the alleles with the two mutations was exactly the same (mutations in haplotype); in the remaining case the difference in the proportion of reads carrying the two mutations very low (<10%).

A total of 18 patients with *EGFR* mutation, but no *KRAS* mutation, were treated in first-line with TKIs: their clinical and *EGFR* molecular status is summarized in Table 2 and S1 Table.

**Table 2:** Molecular and clinical characteristics of patients treated with EGFR-TKIs.

Features	Value	Percentage
**Mean age (years)**	68.4	
**Sex**		
- Male	5	27.8%
- Female	13	72.2%
**Site**		
- Lung	16	88.8%
- Lymph node	1	5.6%
- Pleural effusion	1	5.6%
**Mean % neoplastic cells**	46	
***EGFR* mutation**^a^		
- p.E746_A750delELREA (c.2235_2249del15)	5	27.8%
- p.L747_P753delLREATSP (c.2238_2258del21)	2	11.1%
- p.L747_E749LRE (c.2239_2247del9)	2	11.1%
- p.E746_T751delELREAT (c.2235_2252del18)	1	5.6%
- p.E746_S752delELREATS (c.2236_2256del21)	1	5.6%
- p.L747-A750delLREA (c.2239_2248del10)	1	5.6%
- p.L747_E749LRE (c.2239_2247del9)	1	5.6%
- p.L858R (c.2573T>G)	3	16.7%
- p.E709A/p.G719A^a^ (c.2126A>C / c.2156G>C)	1	5.6%
- p.G719S/p.L861Q^a^ (c.2155G>A / c.2582T>A)	1	5.6%
***EGFR* Exon**		
- 18/18^a^	1	5.6%
- 18/21^a^	1	5.6%
- 19	13	72.2%
- 21	3	16.7%
**Mean % *EGFR* mutated cells**	74.8	
**Response to treatment**		
- Progression Disease	4	22.2%
- Stable Disease	6	33.3%
- Partial Response	8	44.4%
- Complete Response	0	0.0%

^a^ In two patients two *EGFR* mutations were detected. DEL: deletion

### Correlation between the percentage of *EGFR* mutated neoplastic cells and response to TKIs

In the 18 cases treated with EGFR-TKIs (Table 2), the mean of the neoplastic cells in the sample analyzed was 46.1% (range: 5% - 70%) and the median percentage of mutated alleles was 21% (range: 5.0% - 70.0%). The median of mutated neoplastic cells was 74.8% (range: 23.3% - 110%) (S1 Table).

In 6 samples the percentage of mutated cells calculated according to the formula shown above was higher than 100%, reflecting *EGFR* gene amplification with mutant allele-specific imbalance (MASI) [21, 40, 41].

A trend (P = 0.068) was observed between the percentage of mutated alleles and responsiveness to TKIs classified according to the PR>SD>PD scale. No statistical correlation was observed with PFS (P = 0.268) and OS (P = 0.708).

We found a strong correlation (P = 0.017) between the percentage of *EGFR* mutated neoplastic cells and clinical response to TKIs classified according to the same PR>SD>PD scale. No relationship was observed in PFS (P = 0.512) and OS (P = 0.334).

**Fig 2:**
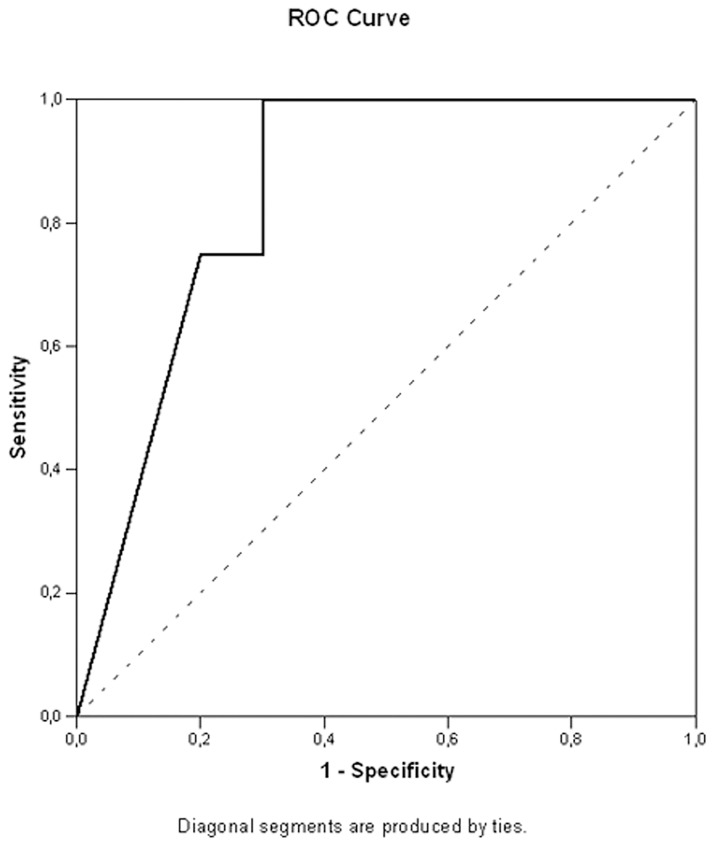
Receiver operating characteristic (ROC) curve examining the relationship between the neoplastic cell percentage and the clinical response to TKIs. Area under the curve (AUC): 0.813, 95% CI 0.603–1.022, P = 0.026. The dotted diagonal line shows the expected ROC curve for a random correlation.

**Fig 3:**
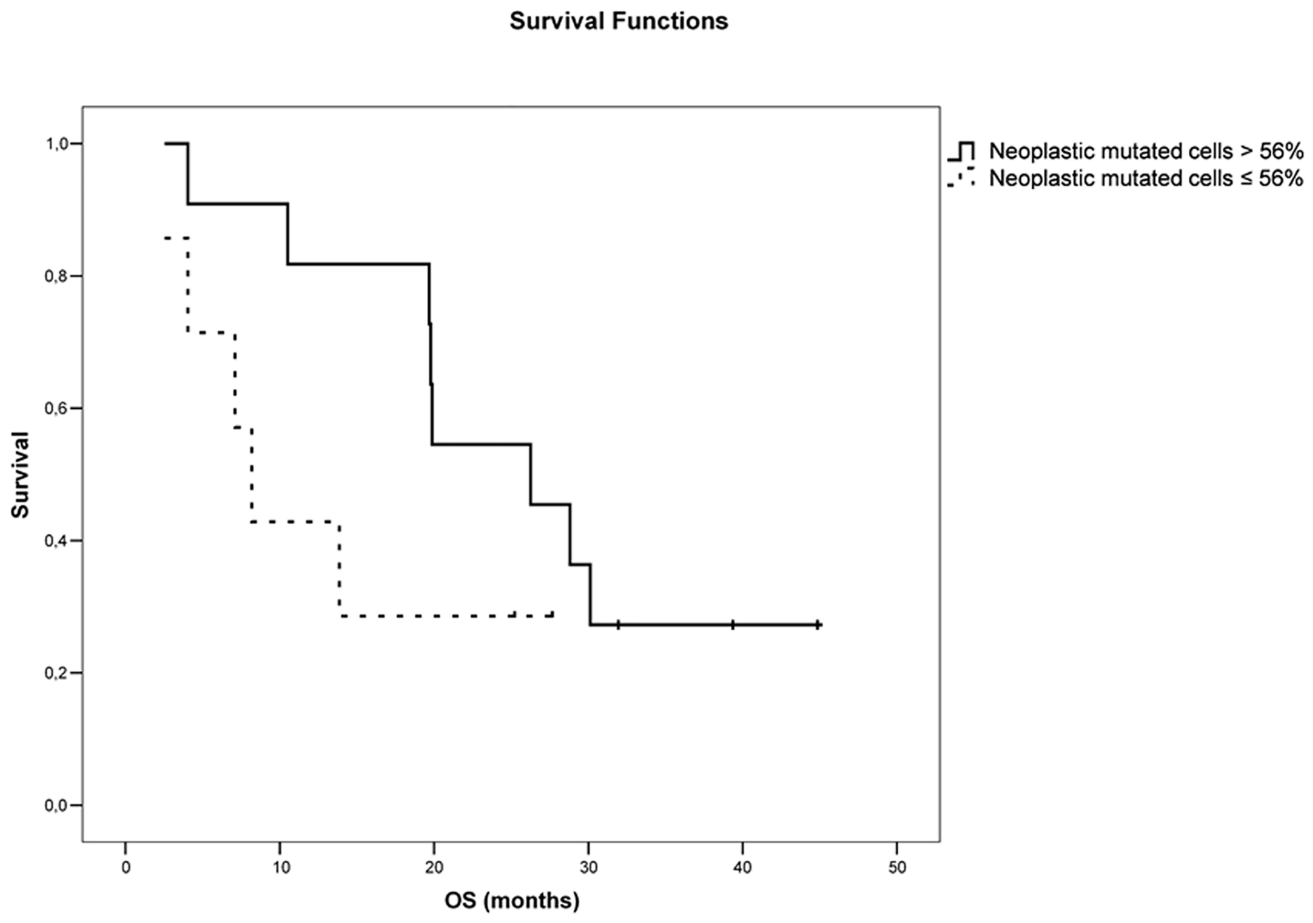
Relationship between Overall Survival (OS) and percentage of mutated neoplastic cells (threshold 56%).

We tried to identify those patients that could benefit most from TKIs therapy. For this, we looked for a threshold of *EGFR* mutated neoplastic cells that could differentiate “responders” (PR group) from “non-responders” (PD/SD group). We calculated and plotted the AUC curve for the percentage of *EGFR* mutated neoplastic cells. A threshold of 56% *EGFR* mutated neoplastic cells was identified as the percentage of neoplastic cells that best correlates with response to TKIs (AUC: 0.813, 95% CI 0.603–1.022, P = 0.026, Fig 2). In fact, patients with a percentage of mutated neoplastic cells equal or lower than 56% had a median OS of 8.2 months (95% CI: 5.4–11.0) compared to the 26.3 months OS (95% CI: 16.5–36.0) of patients with more than 56% mutated neoplastic cells, although the difference did not reach a statistical significance (P = 0.081, Fig 3).

## Discussion

*EGFR* mutations are a key biomarker to select patients with NSCLC for TKI therapy [13–18, 22]. Some studies have noted that the load of *EGFR* mutated alleles associates positively with the response to TKIs [22, 26]. In 2011 Zhou *et al*. observed that the abundance of EGFR mutated alleles correlates with PFS in patients that received gefitinib [22]. Recently, Bria *et al*. in a series of 17 patients, stratified in three groups based on the time of tumor progression, found a statistical trend between the proportion of mutated alleles obtained after NGS mutation analysis and the duration of clinical response to TKIs, no differences were found for PFS and OS [26].

In a similar cohort of patients, we observed only a statistical trend between the percentage of *EGFR* mutated alleles and the clinical response classified as ordinal categories (PR>SD>PD). The correlation was instead very strong (P = 0.017) when the percentage of mutated neoplastic cells was considered as opposed to that of *EGFR* mutated alleles.

In the study mentioned above, Zhou *et al*. did not address the issue of the proportion of *EGFR mutated* neoplastic cells influence on TKI response, but used as cutoff for case selection tumors with >50% neoplastic cells [22]. In our study, we actually attempted to determine the proportion of mutated neoplastic cells that best identifies the subset of patients that benefits most from TKIs therapy: patients with a NSCLC harboring *EGFR* mutations in more than 56% of neoplastic cells have a median OS trice longer in comparison to that of patients harboring tumors with a lower percentage of mutated neoplastic cells. The difference in OS between the two groups falls short of statistical significance (P = 0.081), most likely due to the limited number of the case available for follow up.

Currently, commercial available mutation-specific antibodies allow to detect only two activating EGFR mutations: delE746_A750ELREA and p.L858R. Immunohistochemistry with these antibodies can identify the percentage of mutated neoplastic cells with good specificity, but with highly variable sensitivity (from 40 to 60%) [37]. Therefore, immunohistochemistry with mutation-specific antibodies may be a useful as “screening” method, but cannot replace sequencing of the mutated hot spots.

In approximately one third of our cases the calculated percentage of mutated neoplastic cells was above 100%, most likely reflecting the well-known occurrence of amplification of mutated *EGFR* alleles in lung adenocarcinoma [21, 40, 41]. It should be noted that NGS is not the ideal technique to identify chromosomal gains (i.e. amplification), that are specifically identified by fluorescence in situ hybridization. In our cohort of patients this finding did not influence the clinical response to TKIs (data not shown). This is consistent with a detailed study addressing the issue of *EGFR* mutant allele-specific imbalance in NSCLC that showed how this molecular event does not predict response to TKI therapy [40].

In approximately 15% of our *EGFR* mutated tumors we identified more than one mutation (two *EGFR* mutations or *KRAS* and *EGFR* mutations). The allelic frequency of these concomitant mutations was quite different (data not shown), supporting the hypothesis of different clones in the same tumor, as previously demonstrated in lung cancer [26, 27], as well as in other neoplasms of the thyroid [31, 32], gastrointestinal tract [28], kidney [33] or pancreas [29, 30].

This study indicates that *EGFR* mutation heterogeneity within the tumor is an additional variable that may predict response to TKIs: the percentage of mutated neoplastic cells has an impact on clinical response. Our findings highlight the importance of pre-analytic evaluation of neoplastic material and the use of sensitive quantitative techniques (e.g. NGS technology) for mutation detection. Not only a *“qualitative”* (presence and type of mutation) data, but also *“quantitative”* analysis of the *EGFR* mutational load may be necessary in order to recognize those patients likely to receive the greatest benefit from TKIs treatment.

## Supporting information

S1 Table Molecular characterization of the 18 samples treated with EGFR-TKIs. M, Male; F, Female; DEL, deletion.
(PDF)
