# Supplementary material for: The percentage of Epidermal Growth Factor Receptor (EGFR)-mutated neoplastic cells correlates to response to tyrosine kinase inhibitors in lung adenocarcinoma
Source: PLoS One. 2017 May 16;12(5):e0177822. doi: 10.1371/journal.pone.0177822 (PMC5433779; doi:10.1371/journal.pone.0177822)
Supplement: S1 Table — M, Male; F, Female; DEL, deletion. (PDF) [file pone.0177822.s001.pdf]

Case	Sex	Age	% neoplastic cells in analyzed area	*EGFR* Mutation	Exon	% mutated alleles	% EGFR mutated cells
1	M	47	65	p.E709A	18	32	98.0
				p.G719A	18	32	98.0
2	F	69	20	p.G719S	18	4	40.0
				p.L861Q	21	3	30.0
3	M	63	45	DEL p.E746_A750	19	22	98.0
4	F	46	40	DEL p.E746_A750	19	11	55.0
5	M	81	60	DEL p.E746_A750	19	7	23.0
6	F	82	50	DEL p.E746_A750	19	55	>100
7	F	74	60	DEL p.E746_A750	19	23	77.0
8	F	68	25	DEL p.E746_S752	19	20	>100
9	F	67	45	DEL p.E746_T751	19	20	89.0
10	F	83	30	DEL p.L747_E749	19	5	33.0
11	F	82	50	DEL p.L747_A750	19	14	56.0
12	M	54	30	DEL p.L747_E749	19	70	>100
13	F	60	60	DEL p.L747_E749	19	53	>100
14	M	58	60	DEL p.L747_P753	19	30	100
15	F	86	45	DEL p.L747_P753	19	54	>100
16	F	71	40	p.L858R	21	6	30.0
17	F	76	50	p.L858R	21	13	52.0
18	F	64	50	p.L858R	21	32	>100

S1 Table. Molecular characterization of the 18 samples treated with EGFR-TKIs. M, Male; F, Female; DEL, deletion.
